# miR-125b Upregulates miR-34a and Sequentially Activates Stress Adaption and Cell Death Mechanisms in Multiple Myeloma

**DOI:** 10.1016/j.omtn.2024.102354

**Published:** 2024-10-15

**Authors:** Gabriella Misso, Mayra Rachele Zarone, Angela Lombardi, Anna Grimaldi, Alessia Maria Cossu, Carmela Ferri, Margherita Russo, Daniela Cristina Vuoso, Amalia Luce, Hiromichi Kawasaki, Maria Teresa Di Martino, Antonella Virgilio, Agostino Festa, Aldo Galeone, Giuseppe De Rosa, Carlo Irace, Massimo Donadelli, Alois Necas, Evzen Amler, Pierosandro Tagliaferri, Pierfrancesco Tassone, Michele Caraglia

## Main text

(Molecular Therapy: Nucleic Acids *16*, 391–406; June 2019)

In the originally published version of this article, an image not pertaining to the experimental point “miR-125b-2'-F” was wrongly uploaded in Figure 5D. The authors apologize for any confusion. The correct image is shown below.

**Figure:**
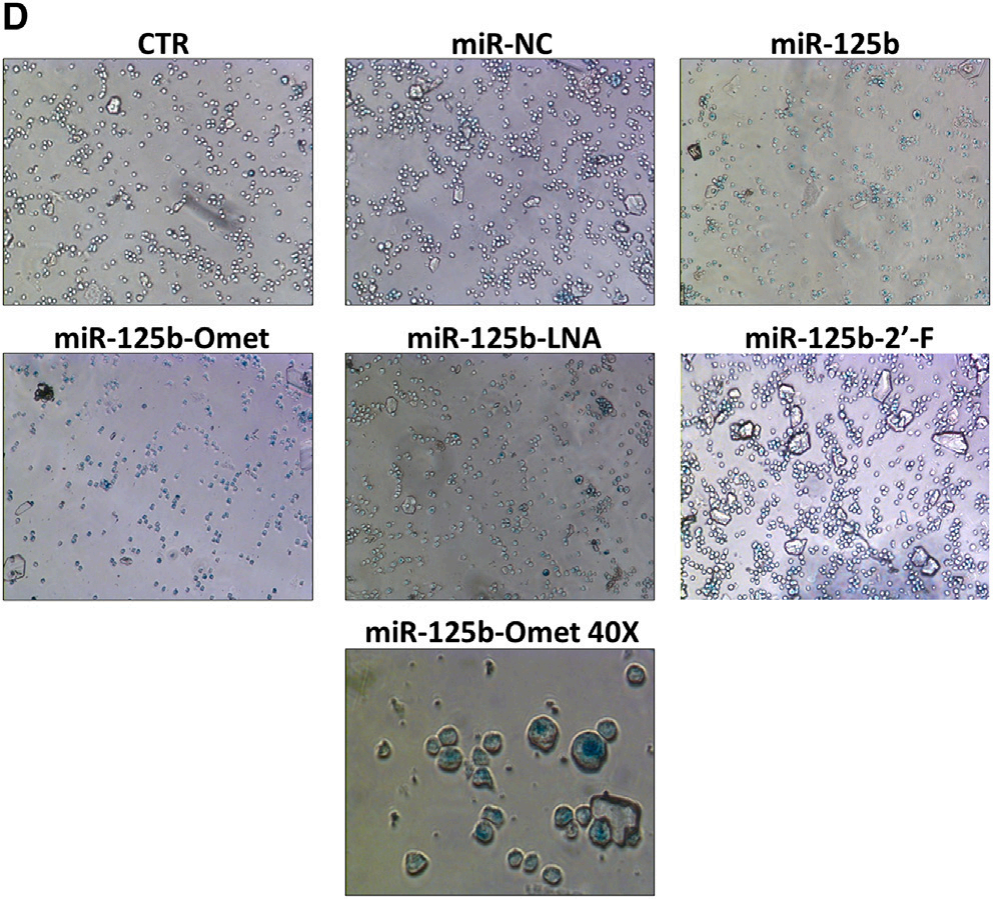
Figure 5D. Analysis of Senescence

